# Shared challenges to the control of complex intracellular neglected pathogens

**DOI:** 10.3389/fpubh.2024.1423420

**Published:** 2024-09-11

**Authors:** Rebecca Lynn Perez, Jemima Chase, Rachel Tanner

**Affiliations:** ^1^Nuffield Department of Clinical Medicine, University of Oxford, Oxford, United Kingdom; ^2^Wadham College, University of Oxford, Oxford, United Kingdom; ^3^Department of Biology, University of Oxford, Oxford, United Kingdom

**Keywords:** tuberculosis, leprosy, leishmaniasis, melioidosis, intracellular pathogens, vaccines

## Abstract

The complex intracellular pathogens *Mycobacterium tuberculosis*, *Mycobacterium leprae*, *Leishmania* spp., and *Burkholderia pseudomallei*, which cause tuberculosis, leprosy, leishmaniasis, and melioidosis respectively, represent major health threats with a significant global burden concentrated in low- and middle-income countries. While these diseases vary in their aetiology, pathology and epidemiology, they share key similarities in the biological and sociodemographic factors influencing their incidence and impact worldwide. In particular, their occurrence in resource-limited settings has important implications for research and development, disease prevalence and associated risk factors, as well as access to diagnostics and therapeutics. In accordance with the vision of the VALIDATE (VAccine deveLopment for complex Intracellular neglecteD pAThogeEns) Network, we consider shared challenges to the effective prevention, diagnosis and treatment of these diseases as shaped by both biological and social factors, illustrating the importance of taking an interdisciplinary approach. We further highlight how a cross-pathogen perspective may provide valuable insights for understanding and addressing challenges to the control of all four pathogens.

## 1 Introduction

**Figure 1:**
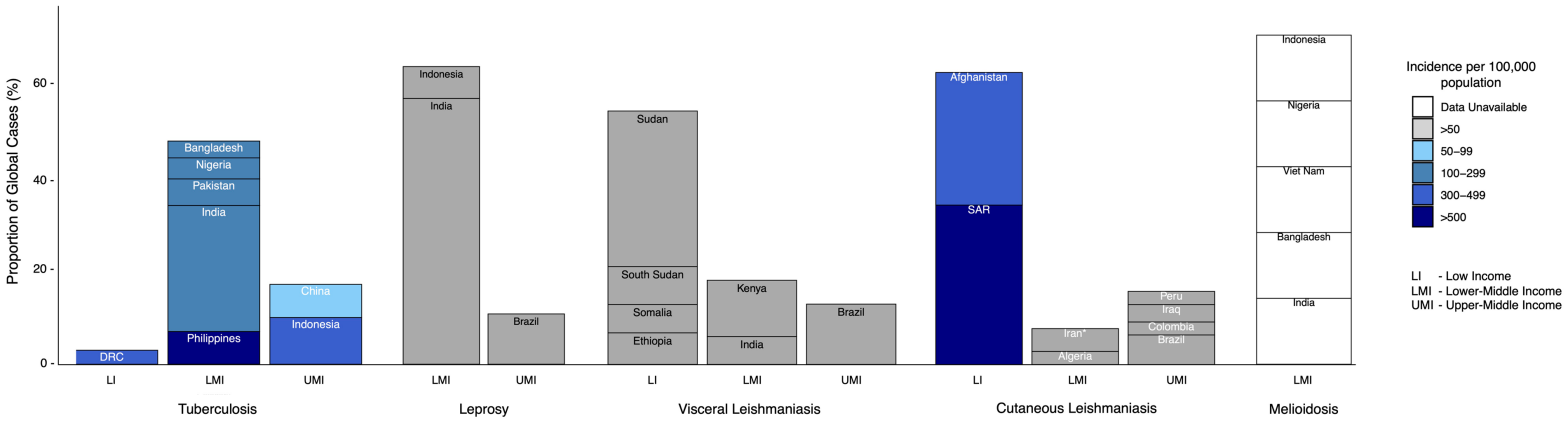
Highest-burden countries (cumulatively making up >70% of the burden of disease) classed by income group (as classified by The World Bank) for each of the exemplar diseases discussed. Color scale reflects estimated incidence per 100,000 population. *Islamic Republic of Iran. SAR = Syrian Arab Republic, DRC = Democratic Republic of the Congo (19, 214–216).

VALIDATE (VAccine deveLopment for complex Intracellular neglecteD pAThogeEns) began as a United Kingdom Research and Innovation (UKRI) Global Challenges Research Fund (GCRF) Network which brings together researchers working on four exemplar complex intracellular neglected pathogens: *Mycobacterium tuberculosis* (*M. tb*), *Mycobacterium leprae* (*M. leprae*), *Leishmania* spp., and *Burkholderia pseudomallei* (*B. pseudomallei*) (1). VALIDATE aims to accelerate vaccine research and clinical development for these pathogens by adopting an innovative integrated cross-pathogen, cross-discipline approach (1, 2). Ostensibly, tuberculosis (TB), leprosy, leishmaniasis and melioidosis do not appear to have many similarities: they differ in their aetiology, pathology and epidemiology among other variances. However, they share important biological and sociodemographic features influencing their incidence, impact and mitigation. These similarities may drive their incidence and persistence in low- and middle-income countries (LMICs; Figure 1) and the gaps that continue to exist in their prevention, diagnosis and treatment. Studying these diseases in conjunction with one another may reveal important commonalities, bolstering understandings of intracellular pathogens and their prevalence in LMICs and inspiring innovations that facilitate control.

**Figure 2:**
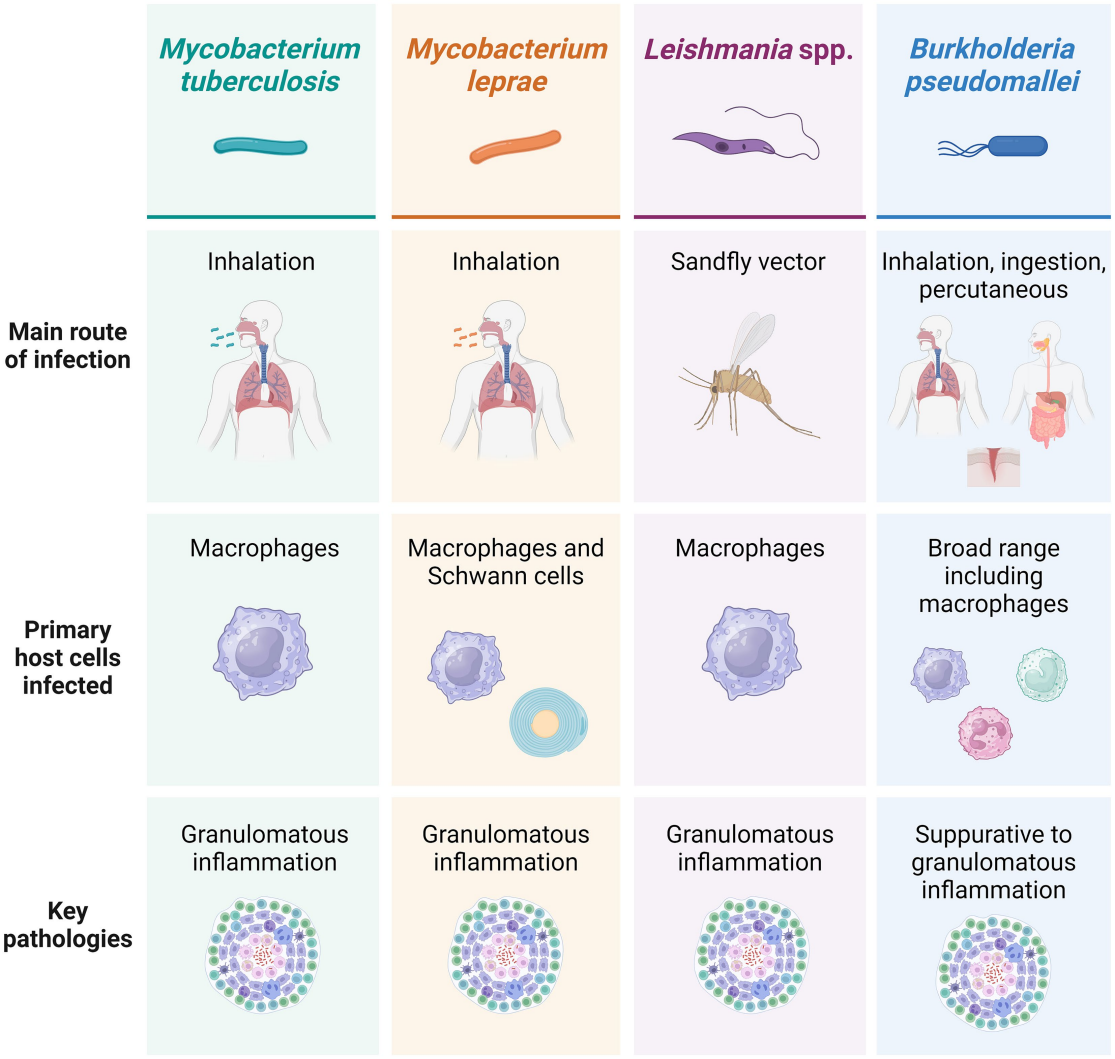
Main infection routes, host cells infected and pathologies associated with each of the four exemplar pathogens discussed. Created using BioRender.

*M. tb*, *M. leprae*, *Leishmania* spp., and *B. pseudomallei* share several key broad characteristics. They are all complex intracellular pathogens, a factor that has important implications for their immunopathogenesis, treatment, and host outcomes. Intracellular pathogens, by definition, establish themselves within host cells, allowing them to evade immune processes such as phagocytosis and antibody neutralization. All four exemplar pathogens demonstrate the ability to survive and replicate within host cells, notably including macrophages (3–6), representing a serious challenge in the elimination of infection and often enabling long-term persistence. They also induce end-stage pathologies with some parallels such as granulomatous inflammation and tissue remodelling (1) (Figure 2). *M. tb*, *M. leprae*, *Leishmania* spp., and *B. pseudomallei* may arguably all be considered ‘neglected’ pathogens. Neglected tropical diseases (NTDs) primarily impact communities that are politically, socially, and economically marginalized and occur in association with substandard housing, lack of safe water, and poor sanitation. As a consequence, they are often under-recognised by national and international health agendas despite the fact that NTDs impact almost 2 billion people (7, 8) and are estimated to contribute to nearly 19 million disability-adjusted life years (DALYs) (9).

Leprosy and leishmaniasis are formally recognised by the World Health Organisation (WHO) and others as NTDs (7), reflecting their burden in impoverished environments and tropical regions. In recent years, leprosy has been largely eliminated as a public health problem in 119 endemic countries. However, it still affects many thousands of people annually, with 174,087 new leprosy cases registered in 2022 (10) (Table 1). This burden is concentrated in a small number of LMICs, with India, Brazil, and Indonesia accounting for 80% of all newly-registered leprosy cases (11). Leishmaniasis is endemic to countries in East Africa, the Eastern Mediterranean region, South-East Asia, and the Americas, and like leprosy, often occurs in rural areas with limited access to treatment. An estimated 700,000 to 1 million new cases of leishmaniasis occur every year (12) (Table 1). In its most serious form [visceral leishmaniasis (VL) or kala-azar], the disease is fatal in over 95% of cases if left untreated (12). The burden of cutaneous leishmaniasis (CL), which may result in visible scarring, is difficult to estimate given the associated social stigma (7), but it is thought that in 2019 an estimated 729,000 DALYs were lost due to cutaneous and visceral leishmaniasis (13).

**Table 1:** Estimated global burden of tuberculosis, leishmaniasis, melioidosis, and leprosy.

Disease	Estimated global prevalence	Estimated incidence	Estimated DALYs (per year)	Deaths (per year)
Tuberculosis	1.8 billion total infections (217)	10.6 million new TB cases/year (218)	65.1 million (219)	1.5 million (220)
Leprosy	165,500 total infections (221)	174, 000 new infections/year (221)	28,800 (219)	N/A
Leishmaniasis	12 million total infections (222)	700,000—1 million new infections/year (223)	729,000 (219)	20,000–30,000 (222)
Melioidosis	Unavailable	165,000 new infections/year (224)	4.6 million (19)	89,000 (224)

All figures represent the most recent available data at time of writing.

TB is not typically defined as a NTD, yet demonstrates important similarities in its occurrence, impact on impoverished populations, and neglect in terms of funding and political will (14). In 2023, an estimated 10.6 million new cases and 1.5 million deaths from TB were reported (15) (Table 1), with incidence highly concentrated in tropical and subtropical LMICs (Figure 1). While more resources may be allocated to TB than other NTDs, the WHO estimated in 2014 that the total resource requirement needed to combat TB and multidrug resistant TB (MDR-TB) for 118 LMICs is 4.8 billion USD each year. Of this, they calculated an unfilled 1.6 billion USD per year gap, representing full treatment for 17 million TB and MDR-TB patients and 6 million lives that could be saved (16). In 2020, funding for TB prevention, diagnosis, treatment, and care was only half of the 13 billion USD target set by the UN Political Declaration on TB (17). Furthermore, there are significant gaps in TB research and technology development, with funding falling short of the estimated required 2 billion USD per year (17) and still no single rapid, accurate, and robust TB diagnostic test suitable for point-of-care use.

Similarly, melioidosis is not yet formally recognized as an NTD by the WHO, although the case has been made for its inclusion as a disease that is ‘almost absent from the global health agenda’, ‘has very limited resources’ and ‘is overlooked by global funding agencies’ (1). Melioidosis is endemic in tropical and subtropical areas, specifically areas of Southeast Asia and northern Australia (18). A systematic review of culture-proven melioidosis cases concluded that melioidosis was responsible for over 4.6 million DALYs in 2015, with mortality (YLLs, years of life lost) accounting for 98.9% of these (19) (Table 1). This burden of disease is higher than that for some recognized NTDs including leishmaniasis and dengue fever (19). Furthermore, prevalence is likely to increase in low-resource contexts, particularly as melioidosis risk is elevated by diabetes mellitus—a disease that is becoming more prevalent in LMICs (20).

The total DALYs associated with NTDs is similar to that of malaria or HIV/AIDS, but lack of consideration of broader disease consequences such as stigma and mental distress results in an underestimation of their burden, potentially disincentivising research and investment (21–23). Visible manifestations of leishmaniasis, leprosy, TB and melioidosis, including lesions, physical deformities and chronic cough, can have significant implications for mental, social, and material wellbeing, such as rejection of employment or ostracization (24–26). Stigmatisation may also influence healthcare-seeking behaviour, present a barrier to timely and effective diagnosis, and negatively impact quality of life.

**Figure 3:**
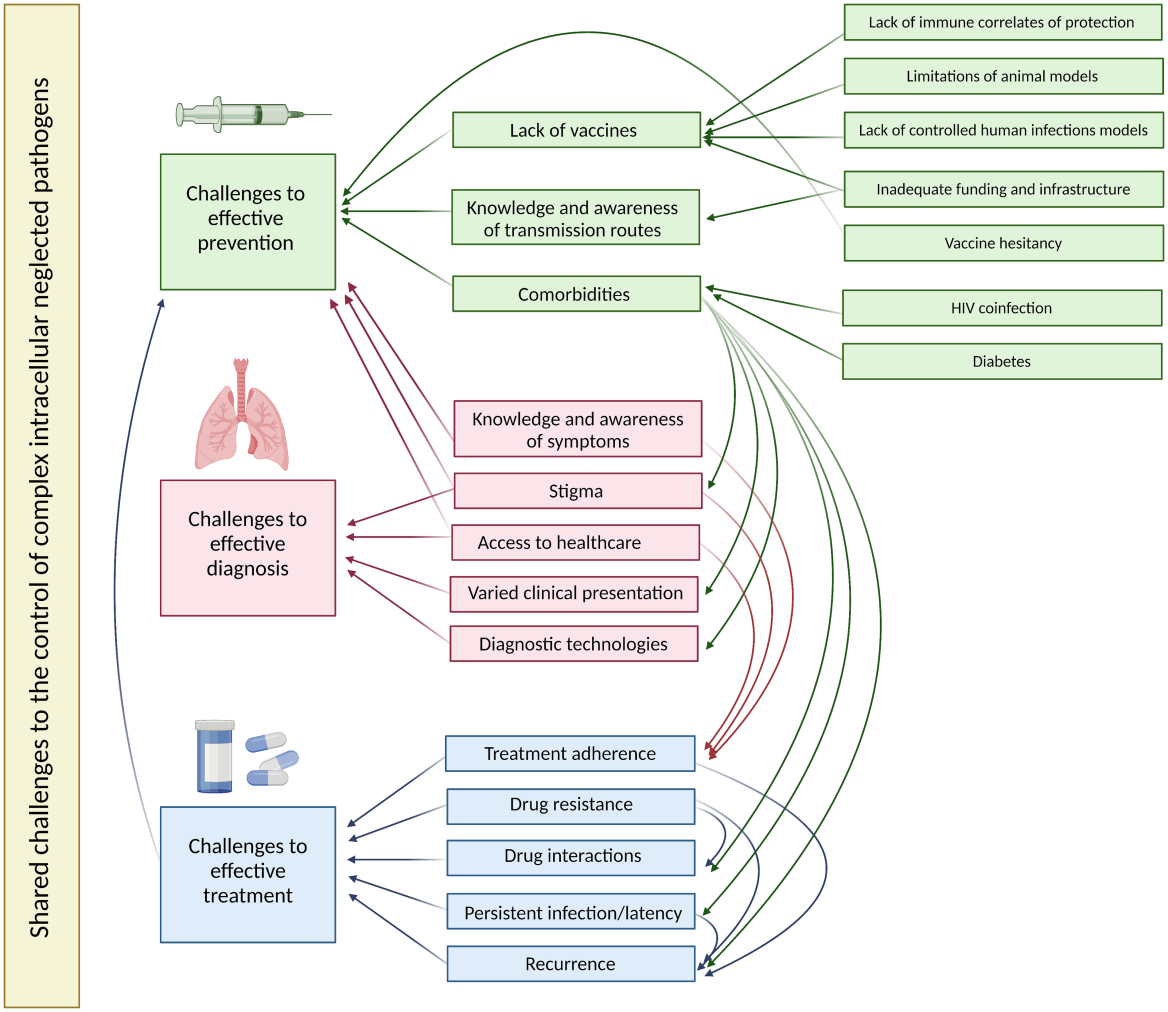
A summary of the shared challenges to effective prevention, diagnosis and treatment of the four exemplar complex intracellular neglected pathogens and their interactions. Created using BioRender.

In line with VALIDATE objectives, we discuss the challenges shared across these four exemplar complex intracellular pathogens and how synergies may be exploited to achieve more effective disease control. Both biological and social issues are addressed, highlighting the value of an interdisciplinary perspective (Figure 3).

## 2 Challenges to effective prevention

### 2.1 Lack of vaccines

Vaccination is widely considered the most effective and economical strategy for controlling any infectious disease. However, the majority of successful vaccines to date are designed to induce humoral immunity against pathogens that are extracellular and/or mediate disease through toxins (27). Immunity against many complex intracellular pathogens requires instead, or in addition, a cell-mediated response, rendering vaccine development far more challenging. Not only must a sufficiently large and persistent pool of antigen-specific memory T cells be induced via a safe delivery system, but these T cells must be of the required phenotype and localized at the correct anatomical site for pathogen clearance (27). While the diseases under discussion may be considered vaccine-preventable based on their natural history, epidemiological data and experimental models, no vaccines against them have yet achieved licensure with the exception of the 100-year old BCG vaccine against TB.

While BCG confers consistent reliable protection against TB meningitis and disseminated disease when administered at birth (28), efficacy against pulmonary disease, the most common form of TB, varies considerably by geographical location and is negligible in many endemic regions (29). As a result of cross-reactivity with conserved antigens, BCG also confers some protection against leprosy, although again estimates vary widely from 20 to 90% and there are concerns around triggering paucibacillary leprosy and neuritis when administered post-exposure (30). Further, live vaccines such as BCG are generally unsuitable for use in immunocompromised individuals (who make up a significant portion of cases, as discussed below) due to risk of disseminated disease (31). A new and more efficacious vaccine against both TB and leprosy is urgently needed.

After decades of neglect, TB vaccine development gained renewed momentum in the 2000s and there are currently 16 vaccine candidates in the clinical pipeline including live attenuated vaccines, inactivated whole-cell mycobacteria, subunit viral vectored vaccines and adjuvanted subunit vaccines (32). Leading candidates including MTBVAC and VPM1002 are in Phase II/III clinical efficacy trials (33). Some TB vaccine candidates also show promise for use against leprosy, such as *Mycobacterium indicus pranii* (MIP) and *Mycobacterium vaccae*. LepVax, a hybrid recombinant protein of four *M. leprae* antigens formulated with GLA-SE as an adjuvant, has advanced to clinical trials (34).

Similarly, several vaccines for leishmaniasis have progressed to clinical evaluation, including ‘first generation’ whole-killed parasite vaccines, live-attenuated parasites and fractionated antigen vaccines which have proven non-efficacious in a prophylactic setting; ‘second generation’ recombinant protein-adjuvant vaccines which have also had limited success; and more promising ‘third generation’ DNA or viral-vectored vaccines such as ChAd63-KH (35). However, the melioidosis vaccine development pipeline lags behind. While a number of live-attenuated vaccine strains and *B. pseudomallei*-derived outer membrane vesicles confer protection in preclinical studies, there are safety concerns and a melioidosis vaccine candidate is yet to progress to clinical testing. Subunit vaccines based on structurally conserved antigens offer safety advantages, and one such candidate, CPS-CRM197/Hcp1, has been shown to confer sterilizing immunity against acute inhalational melioidosis in mice and is due to progress to a phase I clinical trial this year (36). The clinical pipeline for each of the four exemplar pathogens is summarized in Table 2.

**Table 2:** Current clinical trials pipeline for vaccines in development against TB (225), leprosy (43), leishmaniasis (226) and melioidosis (227).

Disease	Vaccine candidate	Vaccine type	Phase
Tuberculosis	AdAg85A	Vectored	I
	TB/Flu04L	Vectored	I
	H107	Subunit	I
	BNT164a1, BNT164b1	RNA	I
	MTBVAC (adolescents/adults)	Live	IIa
	ID93/GLA-SE (QTP101)	Subunit	IIa
	AEC/BC02	Subunit	IIa
	ChAdOx1.85A, MVA85A	Vectored	IIa
	M72 + AS01	Subunit	IIb
	DAR-901	Whole cell	IIb
	BCG revaccination	Live	IIb
	MTBVAC (infants)	Live	III
	VPM1002	Live	III
	MIP	Whole cell	III
	GamTBVac	Subunit	III
Leprosy	LepVax	Protein/adjuvant	I
	*M. habana*	Whole cell	IIa
	*M. vaccae*	Whole cell	IIb
	MIP	Whole cell	III
	ICRC bacilli	Whole cell	III
	Killed *M. leprae*	Whole cell	III
Leishmaniasis	Leish-F1	Second generation	I
	Leish-F3	Second generation	I
	Leish-F2	Second generation	II
	ChAd63-KH	Third generation	II
	Leishvacin	First generation	III
	Autoclaved *Leishmania*	First generation	III
Melioidosis	CPS-CRM197/Hcp1	Subunit	I

Despite this progress, a number of significant and shared challenges remain, including a lack of validated immunological correlates of protection, uncertainty in the predictive value of preclinical animal models, lack of controlled human infection models, and limiting funding for clinical efficacy trials in LMICs.

#### 2.1.1 Lack of validated immunological correlates of protection

To date, no comprehensive immune biomarkers or correlates of protection have been identified for any of the four diseases discussed. A better understanding of which aspects of the immune response mediate protection would greatly facilitate the rational design, optimization and evaluation of vaccine candidates. A correlate would allow vaccine candidates to be down-selected prior to entry into clinical trials and biomarkers could be used as an alternative to clinical disease endpoints, thus shortening trials and expediting vaccine development. However, the fields are embroiled in a catch-22 whereby potential correlates of protective immunity can only be validated in clinical trials when a highly-effective vaccine is developed, yet the design and evaluation of vaccine candidates is extremely difficult in the absence of a validated correlate. For such complex intracellular pathogens, it may be the case that a ‘biosignature’ comprising a combination of immune measures rather than any single parameter will be a more successful correlate. Recent advances in systems technologies such as transcriptomics, proteomics and metabolomics may lead to deeper, more holistic insights (37).

Correlates of protection from *M. tb* infection or TB disease have been extensively investigated. It is now well-established that IFN-γ secreting CD4+ T cells are essential but not sufficient for protection from TB, and this is the most commonly employed measure of immunogenicity in preclinical and clinical vaccine trials (38). However, boosting BCG with the subunit vaccine candidate MVA85A failed to confer enhanced protection over BCG alone despite generating a higher level of these cells, suggesting that we need to look beyond this paradigm (39). Previously under-explored immune parameters including unconventional T cells, Th17 cells, B cells and antibodies are now attracting attention, although their exact contribution to protective immunity and potential as correlates of protection remain ill-defined (38). Recent advances in the TB field are providing novel opportunities for correlate of protection analysis, including the development of *in vivo* controlled human infection models (CHIMs) and *ex vivo* functional mycobacterial growth inhibition assays (MGIAs) (40–42).

Similarly to *M. tb*, Th1 immunity is widely considered to be key in controlling the closely-related *M. leprae*. Indeed, the presence of Th1 cytokines in lesions or lepromin skin reactions is associated with better clinical prognosis and localized rather than disseminated disease (43). However, as is also the case for TB, the limited success achieved by current leprosy vaccines designed to induce Th1 immunity suggests the requirement for other cell types in mediating protection, and a number of candidates have been proposed (44).

Protection against leishmaniasis is complicated by the presence of an intermediate insect vector, particularly as parasite transmission by sand fly bite introduces saliva composed of pharmacologically-active molecules with anti-haemostatic and immunomodulatory potentials (45). It has been shown in mice that the main immune factors responsible for protection after sand fly challenge are rapidly-recruited IFN-γ producing Ly6C+ effector T cells and tissue-resident memory T cells. However, Ly6C is not a human cell marker so the counterparts will need to be characterized in asymptomatic or self-healing individuals (45). Correlates of protection against melioidosis are poorly understood, although IFN-γ responses from CD4+ and CD8+ T cells are again considered essential; IFN-γ-producing NK and NKT cells and humoral immunity may also contribute (46).

#### 2.1.2 Limitations of animal models

The value of preclinical models of TB in predicting vaccine efficacy in humans remains unclear. Indeed, the protection conferred by the candidate TB vaccine MVA85A across preclinical studies did not translate into efficacy in humans (47). An artificial aerosol challenge differs significantly from natural transmission in humans, and the definition of vaccine efficacy differs wildly between preclinical and clinical studies (47). BALB/c and C57BL/6 mice are by far the most popular models for screening of TB vaccine candidates (48)—however, *M. tb* infection in these models does not induce caseous granuloma formation (the hallmark of human disease) and the immune system bears discrepancies with humans in both innate and adaptive features. Guinea pigs are considered a more stringent model but it is difficult to improve upon protection conferred by BCG vaccination. Cattle are a natural target species for TB, and infection with *M. bovis*, which is closely-related to *M. tb*, offers a wide spectrum of TB disease that resembles that found in humans. However, cattle are difficult and costly to maintain under laboratory conditions and lack the cavitations seen in infected humans. Non-human primates are the closest to humans in terms of pathophysiology and are generally considered the gatekeeper for vaccine candidates to progress to human trials; however there are clear ethical and financial constraints to their widespread use.

Despite being one of the first human bacterial pathogens identified, *M. leprae* has not yet been cultured *in vitro*, and it wasn’t until 1960 that the infection was successfully transmitted to animals experimentally. Established and accepted animal models for *M. leprae* remain limited to the mouse plantar footpad cushion model and the nine-banded armadillo. While the mouse footpad lacks nerve involvement, armadillo models demonstrate ability to mimic human disease including extensive neurological involvement (49), offering a potential opportunity to assess vaccine efficacy across the disease spectrum (50). With the increasing availability of mouse knock-out (KO) models and armadillo reagents, the utilization of animal models for leprosy vaccine development is increasingly feasible. Nonetheless, challenges remain (49).

Several animal models have been employed as experimental hosts for CL and VL in vaccine studies—however, to date none satisfactorily reproduces the disease in humans. Guinea pigs and inbred mice are predominantly used as models of CL, and these can mimic the spectrum of disease manifestations observed in human leishmaniasis to some extent (although extrapolation to humans should proceed with caution (51)). For VL, the golden hamster model has been largely superseded by inbred mouse models. However, amastigotes must be injected intravenously in order to induce a reproducible pattern of colonization of the liver and spleen, deviating from natural infection routes. The model further lacks the wasting seen in human disease, and infection is chronic but not fatal. VL induced in BALB/c mice by *L. major* has been proposed as a better model of human VL. The disease pattern in dogs is more similar to humans, and dog reagents are becoming more widely available. As such this may represent the best animal model for vaccine studies, although there are greater ethical implications and dogs have special protections in some countries including the UK.

Evaluation of candidate melioidosis vaccines has been largely restricted to BALB/c and C57BL/6 mouse models. While the former are susceptible to infection and recapitulate acute disease, the latter are more resistant to acute infection and considered most relevant for vaccine testing as animals can attain full protection and sterilizing immunity (52). There is a growing need for well-characterized preclinical models that mimic human infection in order to evaluate vaccine efficacy and safety; in particular an NHP model may be a pre-requisite to advance candidate vaccines to human clinical trials (52).

#### 2.1.3 Lack of controlled human infection models

Development and utilization of controlled human infection models (CHIMs) has greatly accelerated vaccine development for a range of diseases. These models offer the potential to determine efficacy in the target species without the need to wait for long and costly Phase III trials. Instead, vaccine candidates can be rationally down-selected at an early stage of development and correlates of protective immunity identified. However, such models have inherent limitations, including variation in organism preparation and dose and lack of well-defined clinical endpoints. Furthermore, participant safety is paramount, and CHIMs are not ethically feasible where sufficiently attenuated, drug-sensitive strains or effective treatments are unavailable, or where persistent infection or post-infection sequelae are a risk. To date, there are no validated CHIMs for TB, leprosy, melioidosis or leishmaniasis.

A major barrier to developing a CHIM for TB is that it is not ethical to deliberately infect healthy individuals with virulent *M. tb*. However, a live attenuated replicating mycobacterial strain could offer a safe surrogate. In CHIMs based on attenuated *M. bovis* BCG infection by the intradermal route, quantifying mycobacteria from skin biopsies permits detection of a degree of mycobacterial immunity in BCG-vaccinated individuals, but does not mimic the natural route of *M. tb* infection (53–55). Work is now underway to develop aerosol CHIMs using live attenuated *M. bovis* BCG and attenuated strains of *M. tb* that can be reproducibly cleared, are not transmitted, and are detectable and quantifiable *in vivo*. Safety of administering aerosolised BCG has recently been demonstrated (56), but recovery and accurate quantification of bacilli from the airway as a marker of control and therefore vaccine efficacy is challenging (57). A CHIM for leprosy would present even greater ethical and logistical barriers, and to date none has been described. While Hansen and Danielssen attempted to inoculate individuals with nodule tissue, blood and pleural exudate from *M. leprae*-infected patients in the mid-1800s, they were unable to transmit the disease (58). Such experiments pose obvious moral and ethical concerns and indeed, charges were brought against Hansen and he was removed from his duties as a physician (58).

Artificial human infection with *Leishmania* spp. at a discrete site, known as ‘leishmanisation’, has been practiced for centuries in endemic countries as a form of vaccination to protect against lesions on the face and other exposed parts of the body (59). In 2005, the WHO sponsored a large-scale evaluation of the potential for CHIMs as a tool for the evaluation of leishmaniasis vaccine candidates. However, this programme was terminated due to issues with viability of the challenge agent and limited funding opportunities (60). There has been a recent resurgence of interest in a leishmaniasis CHIM as several new candidate vaccines are in the pipeline. However, evidence that some candidate vaccines may protect mice infected via needle inoculation but not those infected by sand fly has highlighted a need to integrate vector transmission into future leishmaniasis CHIMs (60). A first clinical study to evaluate the reproducibility of a CHIM for sand fly-transmitted CL is due to commence shortly. If successful, this could expedite the selection of promising vaccine candidates for progression to clinical trials (61). As treatment of melioidosis requires long-term therapy regimens with no assurance of bacterial clearance, a melioidosis CHIM, even with an attenuated strain, is unlikely to be as feasible.

#### 2.1.4 Inadequate funding and infrastructure for human vaccine trials

It is essential that any vaccine intended for use in LMICs is evaluated in populations and settings representative of those in which it will eventually be deployed. However, the implementation of clinical trial sites for vaccine efficacy testing in LMICs poses major economic and logistical challenges. A 2018 study found that ~83% of clinical trials had been conducted in 25 high-income countries (HICs), whereas <5% were in 91 LMICs (62). Governments in LMICs allocate limited funding to research and the majority comes from HIC-based organisations. Unfortunately, investing in the development and production of vaccines against diseases that primarily burden LMICs is often unattractive to industry due to limited potential for financial profit. LMICs may also lack the skilled personnel necessary to conduct such trials due to a lack of research-based higher education institutions and lack of focus on clinical trials research in medical school curricula. Highly-qualified and experienced individuals often seek work opportunities abroad, resulting in a so-called ‘brain drain’ in their country of origin (63).

Requirements also include a robust clinical and laboratory infrastructure, established field resources and known epidemiological data with which to power trials (64). Currently, very few sites worldwide fulfil these criteria. The first LMIC site developed specifically for the evaluation of TB vaccines was the South African TB Vaccine Initiative (SATVI) site in Worcester, South Africa run jointly by the University of Cape Town. This was followed by a site at the Kenya Medical Research Institute/CDC field station. However, trials need to extend beyond the African subcontinent, particularly those for melioidosis, which is endemic to South East Asia. Ethical approval procedures, a fear of exploitation, complex government regulatory systems and administrative issues can also impede progress (65). Global, cross-continent collaboration is key to addressing this issue, and trial designs and communication must be sensitive to specific cultural and religious contexts. Significant investment in infrastructure and government-implemented changes in approval and regulatory processes are also required. However, there is reason to be optimistic; there has been an increase in clinical vaccine studies conducted in LMICs in recent years. Academic partnerships such as the European and Developing Countries Clinical Trials Partnership (EDCTP) and the former Aeras Global Tuberculosis Vaccine Foundation represent steps toward filling this gap.

#### 2.1.5 Vaccine hesitancy

Vaccine hesitancy is known to be prevalent across a range of socioeconomic, cultural, ethnic and religious backgrounds, but remains understudied in LMICs. While acceptance of vaccinations is higher in LMICs than in Western Europe or the US, scepticism and concern has increased in recent years, apparently exacerbated by misinformation diffused by social media (66, 67). Vaccine hesitancy may pose barriers to both vaccine candidate evaluation and vaccination campaigns. Researchers, governments and global agencies should proceed with care in the case of LMIC-based trials, ensuring effective communication to build trust and avoid triggering vaccine hesitancy (67). Simas and Larson suggest that to address vaccine hesitancy in LMICs, healthcare professionals should be trained accordingly, emerging vaccine confidence crises should be addressed early, communication and outreach strategies should be tailored to the relevant groups/vaccines, and politicians and health authorities should refrain from politicising vaccine debates (66). They emphasize that successful strategies require a tailored approach with consideration of regional, cultural and economic factors (66).

### 2.2 Increased vulnerability due to comorbidity

Comorbidities frequently occur with diseases that result in reduced or altered immune function. Understanding exactly how pathogen infection co-occurs with and influences comorbidities, and in turn how these conditions influence TB, leprosy, leishmaniasis, and melioidosis, is a key need in reducing the burden of mortality from these diseases. In particular, the prevalence of HIV and diabetes in endemic regions raises concerns of syndemics.

#### 2.2.1 HIV coinfection

The burden of HIV/AIDS is focussed in LMICs, and HIV and *M.tb* are closely intertwined, with extensive overlap in the epidemics and coinfection implicated in a large proportion of deaths from both diseases (68). Untreated HIV infection results in the depletion of CD4+ T cells, which play a key role in adaptive immunity against TB (69, 70). Indeed in preclinical models, the absence or depletion of CD4+ T cells results in reduced control of *M. tb* growth in the lungs and accelerated death (71). Despite the relatedness between *M. tb* and *M. leprae*, it seems that HIV infection does not influence the pathogenesis of leprosy in the same way, although disagreement persists within the literature (72–74).

HIV-*Leishmania* spp. coinfection presents a major challenge for VL control (75), and is associated with more severe and unusual clinical manifestations of VL as well as an altered immune response (76, 77). HIV and *Leishmania* share common pathological mechanisms (involving dendritic cells and macrophages), resulting in accelerated progression of both diseases due to increased pathogen replication (78). This does not appear to be the case for melioidosis; the limited studies reported generally observe little difference in disease severity or outcome associated with HIV coinfection (79). Individuals with depleted CD4+ T cell counts retain the capacity for *B. pseudomallei* control, particularly in early stages of the disease (80). Understanding why these differences occur may provide insight into the mechanisms of pathogenesis in immunocompromised patients and offer insight into effectively treating coinfected individuals.

#### 2.2.2 Diabetes

The incidence of Type 2 Diabetes Mellitus (T2D) is rapidly increasing, with 80% of the burden now occurring in LMICs (81). Patients with T2D, particularly those with poor glycaemic control, have elevated numbers of circulating leukocytes that express high levels of inflammatory gene products (82). B cell functioning is impaired in individuals with poor glycaemic control, as elevated levels of blood glucose may modify the structure and function of immunoglobulins (83–85), and T cell functioning also appears to be altered (86–89).

In TB-endemic areas, T2D patients are at elevated risk of developing active TB, experiencing increased disease severity with poorer treatment outcomes compared to normoglycemic individuals (68, 90–92). In recent years, some countries have reported 40–50% incidence of T2D among patients with TB (93–95). The mechanisms by which T2D contributes to TB susceptibility are not fully understood. Several studies have found that patients with T2D have dysregulated immune responses to *M. tb* infection, including an increased inflammatory response (90, 96, 97). The effects of T2D comorbidity in the context of leprosy require greater elucidation, although a higher incidence of diabetes has been reported among lepromatous leprosy patients compared with healthy controls in India and Kuwait (98, 99).

T2D patients are also unusually susceptible to melioidosis infection, with several studies finding an elevated risk of coinfection (100, 101). Diabetic mice have a reduced ability to contain *B. pseudomallei* at the site of infection and reduced capacity to control the inflection once disseminated (102). Inflammatory response dysfunction (89, 103) and decreased cytokine expression (104) in T2D patients may influence this association. Additionally, studies have found that patients with T2D and *M. tb* or *B. pseudomallei* coinfection fail to produce IL-12, resulting in elevated intracellular bacterial loads and poor bacterial killing (105). Effects of TD2 on leishmania are less clear, although it has recently been shown that mononuclear cells from diabetic patients are more susceptible to *Leishmania amazonensis* infection and fail to generate microbicidal molecules (106). Given the impact of diabetes on the immune response, clinicians should also be aware of potential aggravation or unusual presentation of these conditions in the case of coinfection (99, 107, 108).

#### 2.2.3 Sociodemographic risk factors for coinfection

This high incidence of comorbidities is influenced not only by increased biological vulnerability (as a result of altered immune function) but also by factors which elevate general disease risk, including poverty, poor access to medical information or treatment, and overcrowded living situations. For example, the incidence of T2D and diseases such as TB are both impacted by sociodemographic factors such as globalisation, urbanisation, and migration (109). Similarly, stigma, social barriers to testing and treatment, and low-resource healthcare settings and health literacy have contributed significantly to the high incidence of HIV-TB coinfection (110, 111). Understanding the multifactorial landscape influencing the development and treatment of coinfection is a crucial step in addressing comorbidities and reducing the burden of infection and severity of disease.

### 2.3 Knowledge and awareness

Public knowledge of TB, leprosy, leishmaniasis and melioidosis, while variable across and within endemic countries, is generally considered insufficient and may reflect socioeconomic inequalities. Lack of awareness of the clinical signs of these diseases and how they are transmitted limits the extent to which infected individuals can take appropriate measures to prevent their further spread.

While general awareness of TB in endemic areas may be higher than for other diseases, knowledge around transmission is often variable and associated with socioeconomic factors (112). A survey of almost 200,000 adults in India found that, while 89.3% had heard of TB; of those only 55.5% were aware of the mode of transmission (113). In another India-based study, only 52.5% of TB patients were aware that cough was a symptom of TB and 67.2% knew that TB was communicable (114). In a leprosy-endemic community in India, only 26–44% of individuals were reported to understand the cause of leprosy, with up to 28% possessing correct knowledge of transmission and only 16% possessing knowledge of symptoms. While healthcare workers were found have relatively greater awareness, this was still inadequate (115).

Similarly, insufficient awareness regarding leishmaniasis vectors, transmission, risk factors and prevention has been found across endemic areas, including in Saudi Arabia (116); Iran (117); Ethiopia (118); and Morocco (119). Lack of awareness of clinical signs of leishmaniasis has been found to contribute to unreported cases and potentially promote infection reservoirs within families and neighbourhoods (118, 120, 121). Melioidosis awareness in particular remains low in many endemic countries. In Thailand, for example, recent studies suggest that between 74 and 92% of lay adults had never heard of the disease (122, 123). Information on melioidosis and its prevention is not taught in schools and rarely provided by mass media (122). Stigmatisation of these diseases (as discussed further in Section 3.5) may influence public discourse and the willingness of individuals to disclose a suspected infection or seek information.

In addition, with increasing access to social media in LMICs, misinformation around infectious disease will inevitably spread faster and more widely. The onslaught of misinformation that has accompanied the COVID-19 pandemic and consequent mistrust in scientific and medical expertise has highlighted the urgent need to better understand and respond to health misinformation in the modern world (124).

## 3 Challenges to effective diagnosis

Under-diagnosis and delayed diagnosis represent serious concerns for addressing the burden of TB, leprosy, leishmaniasis and melioidosis. Timely case identification is often crucial for limiting morbidity and further transmission of these diseases and for the opportunity to treat. There are common biological challenges including variable clinical presentation and limitations of current diagnostic technologies, but social factors such as limited access to healthcare, lack of knowledge and awareness of disease symptoms, and stigma also play a significant role, particularly in the LMIC contexts in which these diseases most often occur.

### 3.1 Access to healthcare

At the national level, LMICs have less access to health services than their higher income counterparts (125), and access is highly variable across endemic contexts. In rural contexts in particular, healthcare facilities may be located a long distance from patients’ homes and difficult to reach via available transportation (126). In addition, financial and/or temporal costs (such as long wait-times) may impede access (127). In some cases, this may lead to individuals (due to cost, familiarity, distance, or other factors) utilising traditional medicine as a first port-of-call, delaying time from symptom onset to diagnosis (128).

### 3.2 Variability in clinical presentation

All four diseases vary in their presentation, may present years after initial exposure, and share symptoms with other conditions, hindering diagnosis. In most TB-endemic settings, identification of TB cases relies on passive case-finding (waiting for symptomatic patients to seek healthcare). This approach likely misses many cases, particularly among vulnerable groups who face barriers to healthcare access. In 2016, 10.4 million cases of TB were estimated, however, only 6.3 million were reported, indicating high rates of under-diagnosis and/or under-reporting (129). Recognising symptoms of TB—continuous cough, fever, weight loss—may be difficult, as they overlap significantly with other conditions (130). Further, symptoms may manifest differently in cases of extra-pulmonary TB or among children or HIV-positive individuals. Mis- or delayed diagnosis is also a common challenge in the treatment of leprosy. Leprosy displays a wide range of presentations and symptoms typically take 2–6 years to manifest. It is particularly problematic that during subclinical infection, the host may transmit the bacteria and infect others (131).

Manifestations of leishmaniasis vary significantly across visceral, mucocutaneous, and cutaneous forms. Clinical features of leishmaniasis, such as fever and weight loss, may resemble other diseases such as TB, typhoid, and malaria (132). Due to the toxicity of anti-leishmanial drugs, diagnosis is crucial before initiating treatment (133). In the case of melioidosis, there is no pathognomonic feature specific to the disease diagnosis. It demonstrates a wide range of clinical presentations and may similarly be misdiagnosed or mistreated as another infection (134). Further, symptoms may occur several years after initial exposure, obscuring the causative agent. Early diagnosis and appropriate management are crucial in reducing serious complications. Thus, awareness of the varied presentations of these diseases among primary care providers and active case finding are important for addressing the burden of disease.

Diagnosis may also be impeded by comorbidities, which may alter clinical presentation and interfere with immune-based diagnostic assays. This is particularly pertinent given the burden of HIV and diabetes in endemic regions discussed above (75, 76, 97, 99, 135–137). Given that early diagnosis is important for effective treatment, the development of accurate and affordable diagnostic methods for patients with an altered immune response is a key need for reducing the high mortality burden among this group.

### 3.3 Limitations of field-applicable diagnostic technologies

Quantitative diagnostic methods are crucial for rapid and accurate diagnosis of these diseases. Despite continued development of improved diagnostic methods in laboratory contexts, point-of-care diagnostics in endemic regions face significant limitations and the routine use of gold-standard diagnostics is often unfeasible.

In many high-burden settings, relatively insensitive sputum smear microscopy with Ziehl-Neelsen staining or tuberculin skin testing (TST) continue to be utilized for the diagnosis of *M. tb* infection (138). However, the TST cannot distinguish between latent infection and active disease, and may indicate false positives in individuals who were previously BCG vaccinated. The more recent interferon-gamma release assays (IGRAs) are faster and more specific, utilising antigens present in *M. tb* but not BCG to avoid cross-reactivity. However, they are more costly and still unable to differentiate between latent and active forms. Fluorescence microscopy and sputum culture may offer a more sensitive alternative in active disease cases, but such techniques can be costly and time-consuming (139). Furthermore, many such tests cannot distinguish between viable and nonviable organisms, between *M. tb* and other mycobacteria, or between drug-susceptible and resistant strains. Diagnosis of active TB may also include chest radiography, but this requires access to X-ray technology, and some sub-populations such as children may not demonstrate typical indicators of TB in chest radiography or may have extrapulmonary disease (140). As noted, HIV-coinfected patients make up a large proportion of active TB cases, and may demonstrate low bacillary concentrations in sputum as a result of lack of pulmonary cavitation; thus, smear microscopy is negative in more than half of patients with HIV-associated TB. Radiography may similarly fail to identify TB in these patients (139).

Diagnosis of leprosy still widely relies on clinical symptoms. It may be facilitated by skin or nerve biopsy and acid fast staining, however, these depend upon trained personnel and access to appropriate lab facilities (141). Serological tests have been developed and may be useful in monitoring treatment efficacy and disease progression, however, they have not been successful in identifying early or latent forms of leprosy (142). Thus, a quantitative, sensitive, and specific method of leprosy diagnosis which can be deployed in low-resource contexts and identify early or subclinical stages of leprosy infection is needed.

Conventional or quantitative PCR from dermal or mucosal samples is considered the gold standard for leishmaniasis diagnosis, demonstrating high sensitivity and specificity (143). However, such analysis necessitates costly equipment, trained personnel, and laboratory facilities that are often unavailable in primary health facilities in endemic areas. Thus, parasitological methods such as microscopy are typically used in point-of-care diagnostics, involving the direct examination of amastigotes in lesion smear samples. Generating these samples (typically bone marrow, splenic aspirates, or liver biopsies) is invasive and may carry risk to the patient (132). Further, the sensitivity of this method depends on the experience of the microscopist and may decrease with disease chronicity (143).

Laboratory confirmation of melioidosis presents significant challenges. Identification of *B. pseudomallei* by PCR is costly and complicated by the high degree of genetic similarity between closely-related species of the Burkholderia genus (134). Culture-based methods are considered the gold standard for point-of-care diagnosis in endemic contexts. This may be achieved by using a rapid immunofluorescent assay or latex agglutination assays in blood culture which utilise a monoclonal antibody that recognizes *B. pseudomallei* capsular polysaccharide (CPS) (144). However, these methods require specialist equipment and personnel. Further, *B. thailandensis* has been found to express a similar capsular polysaccharide, making it difficult to distinguish between species using culture-based methods. Lab-based methods which can better distinguish between these variants, such as matrix-assisted laser desorption/ionization time of flight mass spectrometry, may currently be unfeasible in low-resource contexts (145).

### 3.4 Knowledge and awareness

Limitations around patient knowledge and awareness discussed in Section 2.3, particularly concerning clinical signs and symptoms, also impact healthcare seeking behaviour and perpetuate diagnostic delays (112). Perhaps even more pertinent in impeding effective diagnosis is widespread lack of knowledge and awareness among healthcare professionals. A systematic review of delay in the diagnosis and treatment of TB noted that a majority of studies identified as the direct or underlying problem “a vicious circle of repeated consultations with a multitude of healthcare providers without a correct diagnosis” (126). Three relevant groups of healthcare providers were identified including primary-level government health posts with poorly-trained personnel, private practitioners with low awareness of TB, and unqualified vendors/traditional practitioners (126). Similarly, multiple consultancies are often required to confirm diagnosis of leprosy and VL (127, 146). Being particularly neglected, melioidosis may not even be considered in differential diagnosis in many areas; for example, a five-year retrospective study in Malaysia found that a majority of melioidosis patients did not receive appropriate treatment due to lack of clinical suspicion (147). A review of case reports of melioidosis in India and Bangladesh found that increased awareness among healthcare personnel, particularly those practicing in rural areas, is essential to guide early diagnosis and timely treatment (148).

### 3.5 Stigma as an obstacle to effective diagnosis

Fear of stigmatisation may motivate patients to delay or avoid diagnosis. Across several cultural contexts, TB was commonly assumed to associate with HIV coinfection or result from poverty, contributing to stigmatisation (149–151). In particular, negative attitudes among healthcare workers may disincentivise care-seeking and reinforce stigmatisation within the community and the healthcare system (150). Individuals with symptoms indicative of TB may aim to avoid stigma by seeing private or out-of-town physicians, potentially resulting in diagnostic delay (152). Similarly, patients affected by leprosy may experience hostility and rejection (153, 154), particularly those with visible marks of infection (155). Stigma has been identified as a significant factor in discouraging patients from seeking healthcare and delayed diagnosis of leprosy. In a Brazil-based study, leprosy patients who feared community isolation were 10 times more likely to wait longer before consulting a doctor (156).

In the case of leishmaniasis, cutaneous disease can cause disfiguring lesions and scars, seen in some societies as a mark of low social status or reflective of underlying poverty, exacerbating outward and perceived stigma (157). Garapati et al. found that the median time between appearance of symptoms and first medical consultation in post-kala-azar dermal leishmaniasis in Bihar, India was 285 days—this delay was attributed to both lack of awareness of symptoms and perceived stigma of the disease (24). While symptoms of melioidosis can be more varied and non-specific, patients often have features that overlap with TB such as fever, weight loss and a productive cough – indeed melioidosis has been dubbed ‘the great mimicker’ – indirectly leading to stigmatisation and potential reluctance to seek care (158).

## 4 Challenges to effective treatment

The development and increasing accessibility of effective antimicrobial treatments for TB, leprosy, leishmaniasis and melioidosis has significantly reduced associated morbidity and mortality worldwide. However, a key similarity across these diseases is that they are all endemic in low-resource settings; a factor that challenges development and delivery of gold-standard care. Understanding the trends and patterns influencing how and why sub-optimal treatment practices occur across these four diseases is key to their successful control.

### 4.1 Drug resistance

Reports of circulating clinical isolates of *M. tb*, *M. leprae*, *Leishmania* parasites and *B. pseudomallei* have raised concerns about the development of widespread drug resistance. Emergent drug resistance is a serious concern for any pathogen and especially for these pathogens which carry a high burden of disease. Furthermore, the limited array and accessibility of treatment options means that emerging resistance to any one standard drug could severely hinder the efficacy of current treatment strategies.

Perhaps the most concerning trend is the emergence of drug-resistant *M. tb*. Worldwide in 2019, around 500,000 people developed rifampicin-resistant TB (RR-TTB), of which 78% had multidrug-resistant TB (MDR-TB; defined as resistant to at least isoniazid and rifampicin). 3.3% of new TB cases and 17.7% of previously treated cases were found to have MDR/RR-TB (WHO 2020). Treatment success of MDR-TB remains low (59% globally) (159). Extensively drug-resistant TB (XDR-TB) refers to MDR strains with additional resistance to any fluoroquinolone and to at least one WHO Group A drug (bedaquiline, linezolid); patients with XDR-TB have worse outcomes than those with MDR-TB. Patients infected with strains demonstrating resistance beyond even XDR-TB may be codified as totally-drug resistant of TDR-TB (159). While less prevalent, there have also been reports of drug-resistant strains of *M. leprae*. Currently, standard-of-care treatment for leprosy is a combination therapy of dapsone, rifampicin, and clofazimine (WHO 2018). In a large study of 1,931 cases conducted by a WHO surveillance network, 154 (8.0%) *M. leprae* strains were found with mutations conferring resistance to at least one antibiotic, 24 of which were MDR (160). While the current threat to treatment is low, these findings indicate that caution and surveillance is needed to prevent greater emergence of resistance.

Leishmaniasis has demonstrated a concerning degree of resilience to standard treatment methods, particularly in endemic areas of the Indian Subcontinent (ISC). For many years, pentavalent antimonials were used successfully worldwide for the treatment of leishmaniasis. In the past several decades, widespread resistance to antimonials (161) has prompted a change in recommended treatment to use of miltefosine (MIL) as a first-line treatment in ISC elimination programmes. However, after only a decade of use, the number of monotherapy MIL failures is increasing (162, 163). For VL in Bangladesh, Bhutan, India, and Nepal, the WHO currently recommends combination therapy of liposomal Amphotericin B (AmB) with MIL or with paromomycin (164). Nonetheless, there are also concerns about the potential for emerging resistance to AmB, indicated by reports of resistant clinical isolates (165–167).

Reports of drug resistant strains of *B. pseudomallei* are limited. However, the current standard treatment for melioidosis relies on a very limited array of drugs and emergent resistance to any one of these is a serious theoretical risk. Current recommendations for treatment of melioidosis involve an intensive phase (a 2 weeks course of ceftazidime or meropenem), followed by a 12-week course of high-dose co-trimoxazole with or without doxycycline (168, 169). A small proportion of strains were found to demonstrate resistance to co-trimoxazole in several national studies in India (168), Thailand (170), and Australia (171). However, a clinical isolate from Indonesia was reported to demonstrate resistance to all drugs tested, including cephalosporins (such as ceftazidime) and carbapenems (169) raising concerns of the potential for extensive drug resistance (XDR) in *B. pseudomallei*.

#### 4.1.1 Biological mechanisms of drug resistance

Studying the array of biological mechanisms of resistance to drug treatment across these four pathogens may highlight similarities and offer new targets for the development of therapeutics. For example, clinically significant efflux pumps have been identified in *M. tb*, *M. leprae*, *leishmania* spp., and *B. pseudomallei* (172–175), and several major *M. tb* efflux pumps associated with drug resistance are also found in *M. leprae*, indicating that these may serve as a resistance mechanism in leprosy as well (172). The development of efflux pump inhibitors (EPIs) such as verapamil and thioridazine to be used as an adjunct to anti-TB therapy is currently being explored (176–178), and may inform strategies for preventing resistance in the other pathogens of interest. Similarly, the development of drugs which target shared mechanisms of resistance—such as impermeability of the cell envelope/wall—may represent a more general approach to tackling drug resistance across pathogenic bacteria.

Understanding mechanisms of adaptive resistance may further enable identification of similar strategies used in other pathogens. For example, the Ser315 variant of the catalase-peroxidase KatG in *M. tb* has been shown to enable resistance to isoniazid (a first-line treatment for TB) (179). When an equivalent KatG variant (Ser324) in *B. pseudomallei* was investigated for similar resistance properties, researchers found a similarly reduced reactivity to isoniazid (180). All of the exemplar pathogens have demonstrated remarkable adaptability to selection triggered by antimicrobial agents. Understanding mechanisms of plasticity and adaptive potential may also enable new approaches to preventing the development of drug resistance.

### 4.2 Treatment accessibility and adherence

The general challenges associated with access to healthcare in LMICs at the national, community and individual level outlined in Section 3.1 clearly impact access to timely treatment. Furthermore, the treatment regimens for these diseases share the common features of being prolonged, complex and costly. A recent systematic review and meta-analysis found that half of TB-affected households face catastrophic health expenditure at the 10% cut-off point (181). Similarly, current treatment for leprosy consists of expensive multiple antibiotics in combination, with therapy duration varying from 6 months to a year in paucibacillary and multibacillary leprosy, respectively (182). Drug supply and cost also remain a major barrier to the access of treatment for VL and melioidosis.

Failure of a patient to adhere to the full treatment regimen, such as by premature discontinuation, is a significant factor influencing the successful clearance of infection and development of drug resistance. This is a particular risk for drug treatment courses that have a long duration (most commonly in the eradication phase, when disease symptoms may have abated and patients no longer feel the need to continue treatment) or involve toxic, expensive, or otherwise inaccessible drugs that increase the difficulty or appeal of adherence. In a survey among healthcare workers in Morocco, 47% stated that patients do not adhere to their anti-leishmaniasis treatment regimens (119).

The currently available drugs for the treatment of these diseases often have significant adverse effects, particularly those used in long courses of treatment, ranging from relatively mild (such as nausea or loss of appetite) to severe (such as nephrotoxicity or peripheral neuropathy) (183–186). Even mild side effects may discourage a patient from completing treatment. Thus, a research priority should be the development of a wider array of antibiotic drugs or delivery technologies that are affordable, require shorter treatment duration and improved side-effect profile, thus reducing barriers to access and adherence.

### 4.3 Recurrence

Even when treatment is effective in the first instance, recurrence (resulting from relapse or exogenous reinfection) is a feature of all four diseases; particularly in immunosuppressed patients. A systematic review and meta-analysis found that the risk of recurrent TB after treatment success is substantial (2.26 per 100 person years at risk), with relapse the most frequent form (pooled proportion of relapses 70%); although the proportion of reinfections increased in high burden regions (187). Both relapse and reinfection also occur in leprosy, with whole genome sequencing recently demonstrating that treated and cured leprosy patients who remain in endemic areas can be infected by another strain (188). In leishmaniasis, recurrence of both CL and VL has been reported (189), and in a study of melioidosis in the Northern Territory of Australia, 4.3% of patients who survived the intensive phase of therapy had molecularly confirmed relapse; in Thailand this rate was 9.3% (190). Improved treatments with lower failure and relapse rates together with vaccines to prevent reinfection would significantly reduce the burden of recurrent disease.

### 4.4 Persistent infection and latency

Latent infection, or a sub-clinical state analogous to latency, is another key feature shared across the exemplar pathogens. *M. tb*, *M. leprae*, leishmania spp., and *B. pseudomallei* may all establish periods of “dormancy” after infection, reactivating opportunistically in response to weakened immune capacity of the host or the cessation of antibiotic treatment (18, 191, 192). Studying these organisms in conjunction with one another may facilitate greater understanding of how intracellular pathogens trigger and maintain a ‘latent’ state, illuminating less-well understood mechanisms of persistent infection and relapse, and creating new targets for treatment and prevention of the emergence of drug resistance.

One of the greatest challenges in treating TB and leprosy involves the ability of *M. tb* and *M. leprae*, respectively, to become dormant in response to environmental stressors such as antibiotic exposure. This occurs via the survival of so-called persister cells which, despite being apparently genetically identical to their drug-susceptible counterparts, enter a non-replicative state until the stressor is absent and may create an evolutionary reservoir from which drug-resistant mutants can emerge (193, 194). Such persistence should not be conflated with latent *M. tb* infection, which results from host immune defences rather than antibiotic pressure, but the two phenomena appear to be phenotypically related and may reflect similar physiological states of the organism (195). Growing evidence also supports the existence of persister forms of *Leishmania* spp. (196). Targeting such persisters could reduce the duration of antibiotic treatment and risk of post-treatment relapse, and thus eliminate potential for the development of resistant strains (175).

Less well-understood are the latency periods associated with melioidosis. Melioidosis often presents with long periods of latency before disease becomes clinically apparent, with reactivated bacterial proliferation occurring when cellular immunity is suppressed (hence the high degree of co-occurrence of active melioidosis and diabetes previously discussed). Reported latency periods have ranged from 19 to 29 years (18). While the exact mechanisms and sites of bacterial persistence remain unclear, they may be informed by studying the mechanisms of latency employed by similarly intracellular pathogens.

### 4.5 Drug interactions

Where treatment for these diseases can be accessed, concomitant therapy for common comorbidities (see Section 2.2) may alter the effects of drugs or their metabolism, worsening treatment outcomes. For example, several studies have suggested that diabetes may affect the pharmacokinetics of certain TB drugs (197–200), potentially resulting in lower concentrations or increased risks of toxicity (199). Higher rates of treatment failure are also reported in patients with concomitant HIV infection and diabetes (76, 199). This may in part be associated with an elevated risk of development of drug resistant pathogen strains in immunocompromised patients (109, 201, 202), but increased treatment duration and altered drug pharmacokinetics have also been suggested to play a role (202). Further investigation is needed regarding interactions between antimicrobials and anti-retroviral drugs (taken by HIV-positive patients) (203), as well as anti-inflammatory drugs (such as glyburide, which may be taken by diabetes patients) (204, 205).

### 4.6 Knowledge and awareness

Beliefs that these diseases are incurable are pervasive, as are myths surrounding their treatment. Patients may reject drug therapy due to disbelief of the diagnosis, negative beliefs about treatment efficacy, fear of potential side-effects, mistrust of modern medicine or beliefs in self- or alternative treatment. For example it has been reported that treatment myths such as consuming tortoise meat will cure TB remain prevalent in India based on focus group discussions (206), and only 48% of respondents in a survey conducted in Southern Iran believed that CL could be treated by medicine (117). Several studies have noted a lack of understanding of treatment, particularly the consequences of defaulting on adherence; adherence to TB therapy has been shown to be facilitated when patients understand the importance of completing treatment (207).

Patients taking other western or traditional medicines may be non-adherent due to fears of negative consequences from taking physician-prescribed medication concurrently. A relationship between non-adherence and pregnancy has also been reported, with some patients believing that drugs could be dangerous, ineffective, or have stronger side-effects during pregnancy. Religion and personal motivation have also been noted as important influences on treatment adherence (207). As is the case for effective diagnosis, clinical failures by healthcare professionals resulting from lack of training, clinician inexperience or inadequate resources may further increase the risk of treatment failure. Poor outcomes may result from the prescription of antibiotics at suboptimal doses, failure to identify existing drug resistance (essentially rendering a combination therapy a monotherapy with suboptimal dosage), or addition of a single drug to a failing regimen.

### 4.7 Stigma as an obstacle to effective treatment

Stigma associated with these diseases has been discussed in section 3.5. As a consequence of its detrimental effect on healthcare-seeking behaviour, stigma can also result in delays in obtaining treatment or failure to receive treatment at all. Furthermore, it can influence treatment adherence – even after the start of therapy, patients may drop out of treatment programs through fear of exposure. Indeed, stigma has been suggested as a potential barrier to home- and work-based directly-observed therapy (DOT) due to the attendance of TB nurses (208). Fear of losing work or dismissal may also result in reluctance to seek support from an employer to purchase medication or obtain leave for treatment. TB-related stigma was the most common motivation cited by HIV-infected Tanzanian patients who did not complete isoniazid preventative therapy, and was associated with non-adherence among patients on DOT in Pakistan. Conversely, TB stigma was a predictor of treatment adherence in a Russian study (208).

Stigma remains a serious obstacle to the efficacy of multi-drug treatment for leprosy: negative social consequences are often perceived as so intense that patients are reluctant to seek or accept treatment (25, 153, 209–211). Sermrittirong et al. found that leprosy patients are more likely to seek treatment far from their home in an effort to conceal diagnoses, causing further psychological and economical burden on the patient and reducing likelihood of adherence (212). Garapati and others found that the time between first medical consultation to onset of specific treatment had a median time of 365 days, ranging from 2 days to 15 years, which they believed to be a result of both lack of knowledge and perceived stigma (24). Stigma surrounding melioidosis is less well-studied, but as described the non-specific symptoms that mimic TB may lead to indirect stigmatisation, and thus reluctance to seek care and receive timely treatment. This is of particular relevance as delayed therapy for melioidosis is associated with a very high mortality rate (213).

## 5 Conclusion

Despite clear differences in their aetiologic agents, important commonalities exist in the biological and social factors impacting the prevention, diagnosis, and treatment of TB, leprosy, leishmaniasis, and melioidosis. Synergistic considerations of these diseases may provide valuable insight for disease control. For all four complex intracellular pathogens, their high burden of disease is in large part a consequence of their occurrence in resource-limited contexts and the unique challenges associated with this, including limited access to gold standard diagnostics and treatment, high prevalence of comorbidities, and lack of funding and infrastructure for research and development.

Prevention efforts are impeded by challenges for effective vaccine development, the prevalence of comorbidities and vulnerable sub-groups, and insufficient public knowledge and awareness. Diagnostic efforts are impeded by the limited availability of and access to accurate and effective diagnostic technologies in endemic contexts, limited awareness of symptoms and their array of presentations, and stigma impacting healthcare-seeking behaviour. Finally, treatment strategies are impeded by variation in treatment adherence, the prevalence and development of drug-resistant strains, drug interactions in patients with comorbidities, and the capacity for persistent infection and latency as well as recurrence. These shared challenges highlight the broad contextual as well as biological factors that drive the persistence of these diseases in resource-limited settings. An integrated cross-pathogen interdisciplinary approach through innovative networks such as VALIDATE will undoubtedly inform and expedite future control strategies.
